# Age and multimorbidities as poor prognostic factors for COVID-19 in hemodialysis: a Lebanese national study

**DOI:** 10.1186/s12882-021-02270-9

**Published:** 2021-02-27

**Authors:** Mabel Aoun, Rabab Khalil, Walid Mahfoud, Haytham Fatfat, Line Bou Khalil, Rashad Alameddine, Nabil Afiouni, Issam Ibrahim, Mohamad Hassan, Haytham Zarzour, Ali Jebai, Nina Mourad Khalil, Luay Tawil, Zeina Mechref, Zuhair El Imad, Fadia Chamma, Ayman Khalil, Sandy Zeidan, Balsam El Ghoul, Georges Dahdah, Sarah Mouawad, Hiba Azar, Kamal Abou Chahine, Siba Kallab, Bashir Moawad, Ahmad Fawaz, Joseph Homsi, Carmen Tabaja, Maya Delbani, Rami Kallab, Hassan Hoballah, Wahib Haykal, Najat Fares, Walid Rahal, Wael Mroueh, Mohammed Youssef, Jamale Rizkallah, Ziad Sebaaly, Antoine Dfouni, Norma Ghosn, Nagi Nawfal, Walid Abou Jaoude, Nadine Bassil, Therese Maroun, Nabil Bassil, Chadia Beaini, Boutros Haddad, Elie Moubarak, Houssam Rabah, Amer Attieh, Serge Finianos, Dania Chelala

**Affiliations:** 1 Department of Nephrology, Saint-George Hospital Ajaltoun, Ajaltoun, Lebanon; 2 Faculty of Medicine, Saint-Joseph University, Beirut, Lebanon; 3 Makassed General Hospital, Beirut, Lebanon; 4 Saydet Zghorta Hospital, Zgharta, Lebanon; 5 Mounla Hospital, Tripoli, Lebanon; 6 Mount-Lebanon Hospital, Hazmiyeh, Lebanon; 7 Orange Nassau Hospital, Tripoli, Lebanon; 8 Islamic Hospital, Tripoli, Lebanon; 9 Youssef Akkar Hospital, Halba, Lebanon; 10 Dar Al-Chifae Hospital, Tripoli, Lebanon; 11 Zahraa Hospital, Jnah, Lebanon; 12 Bahman Hospital, Beirut, Lebanon; 13 Siblin Governmental Hospital, Sebline, Lebanon; 14 Ain Wazein Medical Village, Ain Wazein, Lebanon; 15 Serhal Hospital, Beirut, Lebanon; 16 Iklim Hospital, Mazboud, Lebanon; 17 Centre Hospitalier du Nord, Zghorta, Lebanon; 18 Hotel-Dieu de France Hospital, Beirut, Lebanon; 19 Beqaa Hospital, Zahlé, Lebanon; 20 Abou Jaoude Hospital, Jal el Dib, Lebanon; 21 Labib Hospital, Sidon, Lebanon; 22 Khoury Hospital, Zahle, Lebanon; 23 Hammoud Hospital University Medical Center, Sidon, Lebanon; 24 FMC Hospital, Mejdlaya, Lebanon; 25 Sahel Hospital, Haret Hreik, Lebanon; 26 Beit Chabeb Hospital, Beit Chabeb, Lebanon; 27 Saint-Georges Orthodoxe Hospital, Beirut, Lebanon; 28 Rahal Hospital, Akkar, Lebanon; 29 Jabal Amel Hospital, Tyre, Lebanon; 30 Dinnieh Hospital, Dinnieh, Lebanon; 31 Haroun Hospital, Zalka, Lebanon; 32 Centre Hospitalier Universitaire Notre Dame de Secours Hospital, Byblos, Lebanon; 33 Sacre-Coeur Hospital, Baabda, Lebanon; 34 Middle-East Institute of Health, Bsalim, Lebanon; 35 Saint-Joseph Hospital, Dora, Lebanon; 36 Bellevue Medical Center, Mansourieh, Lebanon; 37 Hayek Hospital, Al Hayek, Lebanon

**Keywords:** COVID-19, Hemodialysis, Mortality, Multimorbidities, SARS-CoV-2, National study

## Abstract

**Background:**

Hemodialysis patients with COVID-19 have been reported to be at higher risk for death than the general population. Several prognostic factors have been identified in the studies from Asian, European or American countries. This is the first national Lebanese study assessing the factors associated with SARS-CoV-2 mortality in hemodialysis patients.

**Methods:**

This is an observational study that included all chronic hemodialysis patients in Lebanon who were tested positive for SARS-CoV-2 from 31st March to 1st November 2020. Data on demographics, comorbidities, admission to hospital and outcome were collected retrospectively from the patients’ medical records. A binary logistic regression analysis was performed to assess risk factors for mortality.

**Results:**

A total of 231 patients were included. Mean age was 61.46 ± 13.99 years with a sex ratio of 128 males to 103 females. Around half of the patients were diabetics, 79.2% presented with fever. A total of 115 patients were admitted to the hospital, 59% of them within the first day of diagnosis. Hypoxia was the major reason for hospitalization. Death rate was 23.8% after a median duration of 6 (IQR, 2 to 10) days. Adjusted regression analysis showed a higher risk for death among older patients (odds ratio = 1.038; 95% confidence interval: 1.013, 1.065), patients with heart failure (odds ratio = 4.42; 95% confidence interval: 2.06, 9.49), coronary artery disease (odds ratio = 3.27; 95% confidence interval: 1.69, 6.30), multimorbidities (odds ratio = 1.593; 95% confidence interval: 1.247, 2.036), fever (odds ratio = 6.66; 95% confidence interval: 1.94, 27.81), CRP above 100 mg/L (odds ratio = 4.76; 95% confidence interval: 1.48, 15.30), and pneumonia (odds ratio = 19.18; 95% confidence interval: 6.47, 56.83).

**Conclusions:**

This national study identified older age, coronary artery disease, heart failure, multimorbidities, fever and pneumonia as risk factors for death in patients with COVID-19 on chronic hemodialysis. The death rate was comparable to other countries and estimated at 23.8%.

## Background

On January 7, 2020, a new virus, the Severe Acute Respiratory Syndrome Coronavirus 2 (SARS-CoV-2), was identified in China, and on February 11, 2020, the World Health Organization (WHO) named the disease Coronavirus Disease 2019 (COVID-19) [1]. One month later, the WHO declared it a pandemic and called all countries to “scale up their emergency response mechanisms”, by addressing four key areas: first preparedness and readiness, second detection, protection and treatment, third reducing transmission and fourth innovating and learning [2]. Dialysis facilities worldwide implemented very early their protective policies, even before recommendations were released [3–10]. In Lebanon, a country of 6 million inhabitants with 80 dialysis facilities and 4300 hemodialysis patients [11], healthcare providers and patients received information regarding infection control and prevention, hand hygiene and use of personal protective equipment (PPE). As in other countries, dialysis units followed a triage protocol at their entries. Symptomatic patients presenting with fever and/or diarrhea and/or respiratory symptoms were isolated during their dialysis session and tested with a nasal swab for real-time reverse transcriptase-polymerase chain reaction (rRT-PCR). Facial masks were mandatory for all patients and staff. Visitors were not allowed to enter the unit. Dialysis units were advised to treat SARS-CoV-2 positive patients isolated in an additional shift at the end of the day.

Despite all protective measures, dialysis patients are at high risk for contracting COVID-19, knowing that they have lower immunity than the general population [12] and they are treated in one large common and crowded space. Moreover, during the last 9 months, many reports from all over the world confirmed the high mortality of COVID-19 in the hemodialysis population, the data being mostly collected between February and May 2020. Mortality rates and prognostic factors were not similar in all countries. In the largest Chinese sample from Wuhan, death rate was estimated at 20% [13]. The Paris Region, Japan, the UK Renal Registry, the Scottish Renal Registry, the Belgian Society of Nephrology, Brazil, Germany, Italy, Spain and New York reported death rates in hemodialysis of 27.3, 16% (at 1 week), 12.5, 26.7, 29.6, 27.7, 26.8, 32, 20 and 27% respectively [14–23]. Recently, the largest two studies conducted in Europe, the ERACODA (European Renal Association COVID-19 Database) and the ERA-EDTA Registry reported a death rate after 28 days of follow-up of 25 and 20% respectively [24, 25].

In Lebanon, the first cases of COVID-19 started to emerge in dialysis units starting March 2020. Up to November 1st, 2020, the Lebanese Ministry of Public Health reported 244 cases of positive PCR in hemodialysis patients from 50 dialysis units [11]. However, due to the lack of patient registries in Lebanon, we have little information about factors related to COVID-19 mortality in hemodialysis. Learning from previous experiences and understanding prognostic indicators may help us detect early high-risk patients and improve their outcomes. Therefore, this national study aims to describe the characteristics of hemodialysis patients with COVID-19 in Lebanon and analyze factors related to a higher mortality.

## Methods

### Study setting

This is a national Lebanese study that enrolled all chronic hemodialysis patients infected with SARS-CoV-2. Lebanon has 4300 chronic hemodialysis patients distributed across 5 main governorates: 760 patients in the capital Beirut, 1530 in Mount Lebanon, 960 in the North, 610 in the South and 410 in Beqaa [11]. All Lebanese nephrologists in the 50 dialysis units that were affected by SARS-CoV-2, were contacted to include their patients.

### Study design and participants

This is an observational study that collected data of chronic hemodialysis patients diagnosed with a positive PCR for SARS-CoV-2 in Lebanon between 31st March and 1st November 2020. An excel sheet including all needed information was sent to each nephrologist. Data were retrieved retrospectively from the medical charts of patients. Excel sheets were completed and sent back after the inclusion of outcomes of all patients (death or end of isolation).

### Eligibility criteria

All chronic hemodialysis patients in Lebanon reported to be infected with SARS-CoV-2 and tested positive by PCR were eligible to be included. Exclusion criteria were patients with incomplete information regarding their comorbidities and outcomes.

### Data collection

Variables that were collected included age, sex, smoking (at the time of infection), dialysis vintage, date of positive and negative PCR when available. Nine comorbidities were analyzed and were added up to define the variable “multimorbidities”: obesity, diabetes, hypertension, coronary artery disease, heart failure, cancer, lung disease, history of stroke and dementia. Symptoms like fever, cough, dyspnea, diarrhea and loss of taste and smell were collected. An open-ended question was added regarding other symptoms worth to mention (labeled as “other”). Nephrologists reported whether the contamination was suspected to have occurred within or outside the unit. Other included variables were the presence of pneumonia on a chest X-Ray or CT-Scan, thromboembolic events, laboratory parameters like C-Reactive Protein (CRP), serum albumin, lymphocyte and neutrophil counts. Clinical variables were collected: number of sessions per week during infection, whether the patient experienced hypotension or hypoxia and whether he/she was managed as an out-patient or was admitted to a regular hospital bed or intensive care unit bed, day of admission, intubation, discharge or death. Medications that were recorded included chloroquine or hydroxychloroquine, corticosteroids, anticoagulants or any antiviral agent.

### Statistical analysis

Continuous variables are presented as mean ± standard deviation (SD) if normally distributed and as median and interquartile range (IQR) if data is skewed. Categorical variables are reported as numbers and percentages. Logistic regression analysis was used to assess the risk factors related to death. We first performed a univariate analysis to assess the relation for each factor with the outcome death. Then we tested all variables adjusted to age and sex. The same procedure was followed for the subgroup of patients who were admitted into the hospital. Statistical analysis was performed using SPSS, version 25. A *P*-value of ≤0.05 is considered statistically significant.

### Ethical approval

The study got the approval of the ethics committee of Saint-Joseph University (CEHDF 1739) and it was conducted in agreement with the Helsinki Declaration of 1975. Data was collected anonymously.

## Results

### Demographics, comorbidities, symptoms and laboratory results

A total of 231 patients on chronic hemodialysis from 41 dialysis facilities were included in the analysis. Distribution across governorates was as follows: 91 patients (39.4%) from the North, 26 (11.3%) from Beirut, 12 (5.2%) from Beqaa, 78 (33.8%) from Mount-Lebanon, 24 (10.4%) from the South. A total of 37 patients (16%) were suspected to have contracted the virus inside the dialysis unit.

The demographics, comorbidities and symptoms of these patients are listed in Table 1. Mean age was 61.46 ± 13.99 years with 55.4% males; 48.1% were diabetics; 79.2% presented with fever. All patients had a positive result on the SARS-CoV-2 PCR assay.

**Table 1:** Demographics, comorbidities, symptoms and laboratory results of patients

	Total *N* = 231	Survivors *N* = 176	Non-survivors *N* = 55
**Age, years**			
*Mean ± SD*	61.46 ± 13.99	59.91 ± 14.31	66.42 ± 11.69
*Median (IQR)*	63 (53, 72)	60 (51.25, 71)	66 (60, 75)
**Dialysis vintage, months**			
*Median (IQR)*	36 (16, 72)	36 (16.25, 71.5)	36 (12, 72)
**Sex, M/F, n(%)**	128/103 (55.4/44.6)	96/80 (54.5/45.5)	32/23 (58.2/41.8)
**Smoking, n(%)**	61 (26.4)	46 (26.1)	15 (27.3)
**Hypertension, n(%)**	201 (87)	151 (85.8)	50 (90.9)
**Diabetes, n(%)**	111 (48.1)	79 (44.9)	32 (58.2)
**Obesity, n(%)**	52 (22.5)	41 (23.3)	11 (20)
**Coronary Artery Disease, n(%)**	91 (39.4)	56 (31.8)	35 (63.6)
**Heart failure, n(%)**	38 (16.5)	18 (10.2)	20 (36.4)
**History of stroke, n(%)**	14 (6.1)	7 (4)	7 (12.7)
**Lung disease, n(%)**	26 (11.3)	16 (9.1)	10 (18.2)
**Dementia, n(%)**	6 (2.6)	2 (1.1)	4 (7.3)
**Cancer, n(%)**	21 (9.1)	13 (7.4)	8 (14.5)
**Fever, n(%)**	183 (79.2)	131 (74.4)	52 (94.5)
**Dry cough, n(%)**	150 (64.9)	101 (57.4)	49 (89.1)
**Dyspnea, n(%)**	110 (47.6)	62 (35.2)	48 (87.3)
**Diarrhea, n(%)**	56 (24.2)	45 (25.6)	11 (20)
**Loss of smell and taste, n(%)**	27 (11.7)	24 (13.6)	4 (7.3)
**Other/Fatigue, n(%)**	18 (7.8)	15 (8.5)	3 (5.4)
**Other/Chills, n(%)**	12 (5.2)	12 (6.8)	0
**Other/Lethargy, n(%)**	10 (4.3)	8 (4.5)	2 (3.6)
**Other/Headache, n(%)**	6 (2.6)	6 (3.4)	0
**Asymptomatic, n(%)**	23 (10)	10 (5.7)	0
**CRP mg/L,** *Median (IQR)*	86.5 (35.3, 190)	69 (27, 150)	143 (74, 233)
**Serum Albumin g/L,** *Median (IQR)*	35 (33, 38)	36.4 (33.5, 40)	30 (24, 35.5)
**Neutrophil/Lymphocyte ratio,** *Median (IQR)*	4.8 (2.4, 7.2)	4.8 (2.3, 7.2)	4.8 (3.6, 6.8)

A total of 78 patients had their CRP level measured, the median CRP was 86.5 mg/L (IQR, 35.3 to 190.0). Only 35 patients had a serum albumin result at the time of diagnosis and the median was 35 g/L (IQR, 33 to 38). And 87 patients had a neutrophil/lymphocyte count and the median neutrophil/lymphocyte ratio was 4.8 (IQR, 2.4 to 7.2).

### Hospital admission and treatment

Out of the 231 patients, 115 patients (49.8%) were admitted to the hospital, 59% within the first day of diagnosis with a median duration between diagnosis and admission of 1 (IQR, 1–3) days. Hypoxia was the major reason for hospitalization. Table 2 describes the percentage of confirmed pneumonia, the number of patients admitted to an intensive care unit (ICU) and those who needed intubation. It summarizes also the main therapeutic agents used in admitted and non-admitted patients. The most used antiviral agent was Remdesivir.

**Table 2:** Diagnosis, admission to hospital/ICU, treatment and death

	Total number of patients *N* = 231	Patients admitted to hospital *N* = 115		Patients non-admitted to hospital *N* = 116	
		Survivors *N* = 67	Non-survivors *N* = 48	Survivors *N* = 109	Non-survivors *N* = 7
**Hypoxia, n(%)**	102 (44.2)	39 (58.2)	45 (93.8)	12 (11)	7 (100)
**Pneumonia, n(%)**	123 (53.2)	48 (71.6)	44 (91.6)	25 (22.9)	7 (100)
**Hypotension during dialysis, n(%)**	67 (29)	20 (29.8)	30 (62.5)	12 (11)	5 (71.4)
**Admission to ICU, n(%)**	46 (19.9)	12 (17.9)	34 (70.8)	0	0
**Intubation, n(%)**	30 (13)	4 (5.9)	26 (54.2)	0	0
**Thromboembolic events, n(%)**	6 (2.6)	1 (1.5)	4 (8.3)	0	1
**Anticoagulation, n(%)**	98 (42.4)	44 (65.7)	33 (68.7)	19 (17.4)	2 (28.6)
**Corticosteroids, n(%)**	106 (45.9)	47 (70.1)	35 (72.9)	21 (19.3)	3 (42.8)
**Hydroxychloroquine, n(%)**	11 (4.7)	8 (11.9)	1 (2.1)	2 (1.8)	0
**Antiviral therapy, n(%)**	21 (9.1)	3 (4.5)	13 (27.1)	4 (3.7)	1 (14.3)

Note: *ICU* intensive care unit

### Time to negative PCR and/or end of isolation

For the 176 patients who survived, mean time to end of isolation was 19.61 ± 7.65 days, with a median of 20 days (IQR, 14 to 22). Out of the 176 patients, 148 patients (84%) had a negative PCR before removal of isolation. The time from diagnosis to negative PCR varied between 2 and 52 days, with a mean of 20.48 ± 7.97 days and median of 21 days (IQR 14 to 24.75). The remaining 28 patients were removed from isolation without a PCR test. In this group, time limit from diagnosis to end of isolation varied between 10 and 21 days, with a mean of 15.04 ± 2.63 days, a median of 14 days (IQR, 14 to 14). Among these patients that were removed from isolation at day 14 without a PCR, only one patient relapsed with fatigue and needed a third week before rejoining the normal shift.

### Mortality

Among the 231 infected patients, 55 died (23.8%). The median time to death after diagnosis was 6 days (IQR, 2 to 10) with a minimum of 1 day and a maximum of 40 days. The different risk factors associated with death are listed in Table 3. Age, coronary artery disease, heart failure, multimorbidities, history of stroke, dementia, fever, dyspnea, hypotension during dialysis and a diagnosis of pneumonia were the most significant risk factors for mortality. After adjusting for sex and age, dementia and history of stroke were no more significant.

**Table 3:** Age and sex adjusted regression analysis of risk factors associated with death in the total sample

	Odds Ratio	95% Confidence Interval	***p***-value
**Age**^**a**^**, years**	1.038	1.013, 1.065	0.003
**Sex**			
Male	1.16	0.63, 2.14	0.636
Female (Ref)			
**Dialysis vintage**^**a**^**, months**	1.002	0.997, 1.007	0.446
**Multimorbidities**^**a, b**^ **(0 to 9)**	1.593	1.247, 2.036	< 0.001
**Smoking**			
Yes	1.123	0.56, 2.27	0.747
No (Ref)			
**Diabetes**			
Yes	1.34	0.71, 2.54	0.365
No (Ref)			
**Obesity**			
Yes	0.88	0.41, 1.88	0.745
No (Ref)			
**Heart failure**			
Yes	4.42	2.06, 9.49	< 0.001
No (Ref)			
**Coronary artery disease**			
Yes	3.27	1.69, 6.30	< 0.001
No (Ref)			
**Lung disease**			
Yes	2.03	0.85, 4.86	0.112
No (Ref)			
**Cancer**			
Yes	1.81	0.68, 4.76	0.233
No (Ref)			
**History of stroke**			
Yes	2.71	0.89, 8.28	0.08
No (Ref)			
**Dementia**			
Yes	4.46	0.77, 25.88	0.096
No (Ref)			
**Fever**			
Yes	6.66	1.94, 27.81	0.003
No (Ref)			
**Diarrhea**			
Yes	0.705	0.33, 1.51	0.366
No (Ref)			
**Dyspnea**			
Yes	14.11	5.84, 34.13	< 0.001
No (Ref)			
**Hypotension**			
Yes	8.03	3.94, 16.36	< 0.001
No (Ref)			
**Pneumonia**			
Yes	19.18	6.47, 56.83	< 0.001
No (Ref)			
**C-Reactive Protein**			
> 100 mg/L	4.76	1.48, 15.30	0.009
< 100 mg/L (Ref)			
**Admission to hospital**			
Yes	12.70	5.25, 30.74	< 0.001
No (Ref)			

^a^For continuous variables, the odds ratio refers to the change per unit of a given variable

^b^"Multimorbidities” is a continuous variable and reflects the sum of 9 comorbidities: Hypertension, diabetes, obesity, coronary artery disease, heart failure, history of stroke, lung disease, dementia, cancer

When analyzing the subgroup of admitted patients, the risk factors associated with mortality were similar to the total sample but the lung disease appeared significant in this subgroup (Table 4). There was a trend for highest mortality among men compared to women admitted however not significant. Only one out of 11 patients treated with hydroxychloroquine died. Three of these patients were already on hydroxychloroquine for lupus. The antiviral treatment did not have any beneficial effect on death.

**Table 4:** Age and sex adjusted regression analysis of risk factors associated with death in the subgroup of admitted patients

	Odds Ratio	95% Confidence Interval	***p***-value
**Age**^**a**^**, years**	1.038	1.006, 1.072	0.021
**Sex**			
Male	1.88	0.89, 3.99	0.100
Female (Ref)			
Multimorbidities*^a^ **(0 to 9)**	1.495	1.12, 1.99	0.006
**Diabetes**			
Yes	1.56	0.71, 3.43	0.267
No (Ref)			
**Coronary artery disease**			
Yes	3.51	1.54, 7.97	0.003
No (Ref)			
**Heart failure**			
Yes	3.07	1.18, 7.96	0.021
No (Ref)			
**Lung disease**			
Yes	4.06	1.19, 13.88	0.026
No (Ref)			
**Corticosteroids**			
Yes	1.42	0.56, 3.60	0.460
No (Ref)			
**Antiviral therapy**			
Yes	9.11	2.22, 37.48	0.002**
No (Ref)			

^**a**^For continuous variables, the odds ratio refers to the change per unit of a given variable

*"Multimorbidities” is a continuous variable and reflects the sum of 9 comorbidities: Hypertension, diabetes, obesity, coronary artery disease, heart failure, history of stroke, lung disease, dementia, cancer

**p = 0.013 after adjustment for age, coronary artery disease and heart failure

## Discussion

This is the first national study of hemodialysis patients with COVID-19 in Lebanon. Our results confirm the high mortality rate of this vulnerable population, as described in several previous reports from other countries [14, 17, 19, 20, 22, 23, 26]. Our patients’ death rate was estimated at 23.8%, very close to the 25% reported by the ERACODA study of 26 European and North Mediterranean countries including 768 dialysis patients [24]. To our knowledge, this is the first study that evaluates the number of comorbidities or multimorbidities as a risk factor for death in hemodialysis patients with COVID-19. The nine comorbidities included in our analysis were diabetes, hypertension, obesity, heart failure, coronary artery disease, history of stroke, lung disease, dementia and cancer. When analyzed one by one, only heart failure, coronary artery disease, history of stroke and dementia were found significant risk factors for increased mortality. However, when they were added, every increase in one comorbidity on a scale of 1 to 9, was associated with a 59% more death. Other studies identified one or two of these poor prognostic factors. For instance, in the ERACODA study, obesity was found a risk factor but not diabetes, nor lung disease, nor coronary artery disease [24]. Diabetes had a trend to increase mortality in the French study but did not reach significance [14].

Importantly, in all dialysis and general population’s COVID-19 studies, the most consistent demographic risk factor for death was age. Our study confirmed the significant association of older age and death, which is also aligned with the findings of the European ERACODA study [24]. Age was also identified as a risk factor for higher mortality in studies from the UK Renal Registry, from Japan and Spain [15, 16, 22]. On top of age, the ERA-EDTA study that included 3285 patients found male patients at higher risk for death [25]. In our population, only male patients who were admitted to the hospital showed a non-significant trend to higher mortality compared to females.

Regarding dialysis-related factors, dialysis vintage was not associated with higher death in our series although it was demonstrated to be a risk factor in the 2385 patients from the UK Renal Registry [16]. On the other hand, hypotension during dialysis was a poor prognostic factor in our patients consistent with the results of a study of 108 patients from London [27]. Interestingly our study showed a CRP cutoff above 100 mg/L as a poor prognostic marker. This is aligned with the French study from the Paris region that found an association between a CRP > 175 mg/L and higher mortality [14]. This also concurs well with two studies one from Wuhan, China, and a second one from Turkey that identified a high CRP as predictor for higher mortality [28, 29]. These studies found as well the low neutrophil/lymphocyte ratio as a predictor for death [28]; this was not confirmed in our patients.

In our infected hemodialysis population, 90% of patients were symptomatic. The symptomatology described is consistent with several worldwide reports. Fever is the most frequent symptom, followed by dry cough, dyspnea and to a lesser degree diarrhea [23, 28, 30, 31]. The first reports of COVID-19 in dialysis emphasized the frequency of diarrhea [3] but it was not confirmed in larger samples. In a Spanish case-series, 77% of patients had fever (33% pneumonia), these results are similar in our population [30]. However, in one Turkish and one Chinese series, fever was found in 30 and 51.9% of 42 and 131 cases respectively [13, 28]. Surprisingly, the Turkish patients had more cough and dyspnea than other populations although they had no difference in demographic factors. In series that found higher rate of asymptomatic patients, dialysis units were screened regularly [32]. Screening of dialysis units was performed in our dialysis population only in centers where number of infected patients was high, thus we may have missed several asymptomatic cases.

The 50% rate of admission of our patients was lower than other countries. In the Dutch-speaking Belgian Renal Society patients, 138 out of 228 patients (60%) were admitted [18]. In the French series, 41 out of 44 patients (93%) were admitted although only thirty-three needed oxygen therapy [14]. Despite the difference in admission rates, the death rate was similar. In fact, our results showed that managing these patients on an outpatient basis is possible and safe as long as the patient does not need oxygen therapy.

The decision to end isolation was based on different criteria across the 41 units that took part in this study. A high percentage of our patients needed repeated PCR testing before it became negative. This is in agreement with several previous reports confirming the prolonged shedding of SARS-CoV-2 in hemodialysis patients that can reach sometimes 74 days [23]. Therefore, many are convinced of the importance of repeating PCR testing before ending isolation [33]. However, despite this prolonged shedding, 28 out of the 176 patients that survived were removed from isolation after 14 days without PCR testing. Only one of these 28 patients presented relapsing fatigue leading to a further third-week isolation. The remaining 27 patients did not show any symptoms. Although several reports in the literature recommended not to end isolation without two consecutive negative PCRs, the Center for Disease Control and Prevention (CDC) states that isolation could be ended in asymptomatic patients without confirmation with a negative PCR [10]. This was shown to be uneventful in our population.

Finally, the treatment used in the first diagnosed patients was hydroxychloroquine and did not cause an increase in death as opposed to the study published by the Spanish kidney registry in March 2020 [22]. Hydroxychloroquine was also found safe in a French series of 21 hemodialysis patients [34]. In our extremely ill patients admitted to the ICU, antiviral treatment did not show any benefit regarding mortality but we cannot make further conclusions because it was prescribed in severe cases, which is considered as a bias of indication.

Our study has some limitations. First, data were collected retrospectively leading to some information biases especially regarding symptoms that would have not been documented in medical charts like loss of smell or taste. Second, missing data on neutrophil/lymphocyte ratio for a large number of patients may have underestimated the importance of this possible risk factor. Third, the lack of information about the dose of hydroxychloroquine used cannot lead to definite conclusions regarding the safety of this drug in dialysis patients.

Despite these imitations, the major strength of our study is the representative sample that included almost all hemodialysis patients infected with SARS-CoV-2 in Lebanon during a seven-month period. Although a few centers did not share their data but this study still included 95% of all reported patients. This study highlights the importance of comorbidities as risk factors for mortality in hemodialysis patients with COVID-19.

## Conclusions

In this national study, the death rate among hemodialysis patients with COVID-19 was estimated at 23.8%. This study identified older age, multimorbidities such as coronary artery disease, heart failure, history of stroke and dementia, as well as fever and pneumonia as poor prognostic factors. Rigorous protective measures need to be implemented and followed especially in dialysis patients carrying these high-risk characteristics.

## Data Availability

All data generated during this study are included in the manuscript.

## Abbreviations

COVID-19: Coronavirus Disease 2019
SARS-CoV-2: Severe Acute Respiratory Syndrome Coronavirus 2
HD: Hemodialysis
PCR: Polymerase chain reaction
CRP: C-Reactive Protein
